# Trends in use of prescription stimulants in the United States and Territories, 2006 to 2016

**DOI:** 10.1371/journal.pone.0206100

**Published:** 2018-11-28

**Authors:** Brian J. Piper, Christy L. Ogden, Olapeju M. Simoyan, Daniel Y. Chung, James F. Caggiano, Stephanie D. Nichols, Kenneth L. McCall

**Affiliations:** 1 Department of Medical Education, Geisinger Commonwealth School of Medicine, Scranton, Pennsylvania, United States of America; 2 Department of Addiction Medicine, Geisinger Marworth Alcohol and Chemical Dependency Treatment Center, Waverly, Pennsylvania, United States of America; 3 Department of Pharmacy Practice, Husson University School of Pharmacy, Bangor, Maine, United States of America; 4 Department of Psychiatry, Tufts University School of Medicine, Boston, Massachusetts, United States of America; 5 Department of Pharmacy Practice, University of New England College of Pharmacy, Portland, Maine, United States of America; Chiba Daigaku, JAPAN

## Abstract

**Background:**

Stimulants are considered the first-line treatment for Attention Deficit Hyperactivity Disorder (ADHD) in the US and they are used in other indications. Stimulants are also diverted for non-medical purposes. Ethnic and regional differences in ADHD diagnosis and in stimulant use have been identified in earlier research. The objectives of this report were to examine the pharmacoepidemiological pattern of these controlled substances over the past decade and to conduct a regional analysis.

**Methods:**

Data (drug weights) reported to the US Drug Enforcement Administration’s Automation of Reports and Consolidated Orders System for four stimulants (amphetamine, methylphenidate, lisdexamfetamine, and methamphetamine) were obtained from 2006 to 2016 for Unites States/Territories. Correlations between state level use (mg/person) and Hispanic population were completed.

**Results:**

Amphetamine use increased 2.5 fold from 2006 to 2016 (7.9 to 20.0 tons). Methylphenidate use, at 16.5 tons in 2006, peaked in 2012 (19.4 tons) and subsequently showed a modest decline (18.6 tons in 2016). The consumption per municipality significantly increased 7.6% for amphetamine and 5.5% for lisdexamfetamine but decreased 2.7% for methylphenidate (all *p* < .0005) from 2015 to 2016. Pronounced regional differences were also observed. Lisdexamfetamine use in 2016 was over thirty-fold higher in the Southern US (43.8 mg/person) versus the Territories (1.4 mg/person). Amphetamine use was about one-third lower in the West (48.1 mg/person) relative to the Northeastern (75.4 mg/person, *p* < .05) or the Midwestern (69.9 mg/person, *p* ≤ .005) states. States with larger Hispanic populations had significantly lower methylphenidate (*r*(49) = -0.63), lisdexamfetamine (B, *r*(49) = -0.49), and amphetamine (*r*(49) = -0.43) use.

**Conclusions:**

Total stimulant usage doubled in the last decade. There were dynamic changes but also regional disparities in the use of stimulant medications. Future research is needed to better understand the reasons for the sizable regional and ethnic variations in use of these controlled substances.

## Introduction

The prevalence of Attention Deficit Hyperactivity Disorder (ADHD) increased to 11.0% of US children in 2011 of which two-thirds received pharmacotherapy [1]. This was a 41% increase relative to the prevalence in 2003 [2]. A five-fold difference was found in parent reported ADHD medication use between states. ADHD prevalence among children in San Juan was 17% of that in Hartford [3]. Adult ADHD prevalence was 4.4% [4] and lifetime prevalence was 8.1% [5]. Other variables associated with ADHD medications were male sex and white ethnicity [1,6]. Over three-quarters of the Diagnostic and Statistical Manual of Mental Disorders (DSM-5) working group members for ADHD and disruptive behavior disorders had ties to the pharmaceutical industry [7]. The 2013 DSM-5 criteria for ADHD were more inclusive of adult ADHD [8]. There was a 344% increase in women (15–44) with private insurance that filled an ADHD medication prescription between 2003 and 2015 [9].

There are a variety of pharmacotherapies for ADHD. The stimulants (methylphenidate, amphetamine, methamphetamine, lisdexamfetamine) are the most frequently prescribed [10], are available in different formulations, and are sympathomimetics that increase catecholamine levels (Table 1). Methylphenidate, amphetamine, and methamphetamine show similar potency for the norepinephrine transporter and the potency for the dopamine transporter is similar to that of cocaine [11]. These agents are recommended as first-line by US guidelines [12] although others place a greater emphasis on non-pharmacological treatments [13]. Although amphetamine was US FDA approved for use in preschoolers, the preponderance of agents administered (91.4%) to 3–5 year olds were not FDA approved [14]. Use of ADHD medications was seven-fold more common in 2012 in the US than in the UK [15]. Cochrane meta-analyses have noted that methylphenidate may improve teacher and parent rated ADHD symptoms but that the evidence quality was “very low” with difficulties in clinical trials with participant blinding and outcome assessors to the active substance versus the placebo [16, 17]. Methodological deficiencies have also been documented among adult-ADHD trials including short-duration, limited sample sizes, exclusion of patients with common comorbidities, and difficulties with conflicts of interest disclosure [18]. Improved performance on standardized tests in reading and mathematics was observed among children with ADHD that received medications relative to unmedicated peers [19]. Although methamphetamine is better known as a recreational drug, a rarely used pharmaceutical formulation (Desoxyn) was approved for ADHD.

**Table 1:** Comparison of the US Food and Drug Administration (FDA) approved indications, mechanism of action, Controlled Substance Act Schedule, formulations, half-life, and cost of the drugs.

	amphetamine	methylphenidate	lisdexamfetamine	D-methamphetamine
FDA approved for (age)	ADHD (3–17, adults) narcolepsy (6–17)	ADHD (6–17, adults) narcolepsy (adults)	ADHD (6–17) BED (adults)	ADHD (6–17) obesity (adults)
Pharmacodynamics^1^	DAT, NET, & VMAT	NET & DAT	DAT, NET, & VMAT	DAT, NET, & SERT
US Schedule (UK Class)	II (B)	II (B)	II (B)	II (A)
Dose (mg/day)^2^	≤ 30	≤ 60	30 to 70	20 to 25
Formulations^F^	immediate & extended release	immediate & extended release transdermal	immediate & extended release	immediate release
Half-life (hours)	9.8–11^D^, 11.5–13.8^L^	2^-^, 4^+^	12^D^	4–5
Cost/year Medicaid^3^ (Millions USD)	$449.1	$700.4	$782.7	NR
Cost/year (USD) / patient 9–12 months for Medicaid^3^	$2,241–2,988	$1,764–2,352	$2,016–2,688	NR

BED: Binge Eating Disorder; DAT: dopamine transporter; NET: Norepinephrine Transporter; SERT: serotonin transporter; VMAT: vescicular monoamine transporter.

^-^(-) enantiomer

^+^(+) enantiomer

^D^dextroamphetamine

^L^levoamphetamine

^F^all formulations are reported to the Drug Enforcement Administration

NR: not reported

^1^Stahl, 2013.

^2^package insert

^3^https://www.cms.gov/Research-Statistics-Data-and-Systems/Statistics-Trends-and-Reports/Dashboard/2015-Medicaid-Drug-Spending/2015-Medicaid-Drug-Spending.html

The etiology of ADHD is multifaceted and involves biopsychosocial processes [20–27]. There may be social or economic incentives that encourage an ADHD diagnosis [21, 28, 29]. Public school children diagnosed with ADHD may qualify for additional educational services and adults diagnosed with ADHD may receive additional time for tests. Among young adults, particularly those in higher education, stimulant use may be extremely prevalent [30, 31]. ADHD with documented severe impairments can also result in Supplemental Security Income (SSI). SSI benefits for ADHD began in 1990 and were more common than those for intellectual disability, autistic disorder and other pervasive developmental disorders, and speech and language impairments in 2013 [32].

The origin for the recent increases in ADHD diagnoses [1, 2, 33] in the US is unknown. These elevations were especially pronounced among low-income children [34]. Changes from the DSM IV to DSM-5 criteria including the creation of subtypes may have resulted in the diagnosis of more females [9] and later modifications were more inclusive of preschoolers [35]. Additionally, patients who developed symptoms between the ages of 7 and 11 now meet criteria for ADHD diagnosis. Lastly, the DSM-5 changed the number of required criteria from 6 to 5 for adults and provides examples of how ADHD might manifest in the life of adults, which aids in increasing diagnosis in this patient population. One intriguing hypothesis for the increase is that the *No Child Left Behind* (NCLB) *Act of 2001* indirectly resulted in pressure from US school districts on parents to have low academically performing children evaluated for ADHD, be administered cognitively enhancing drugs, and perform better on standardized tests in math and reading which would prevent the potential loss of school funding [28]. Low-income public school children from states where this consequential accountability was introduced as part of NCLB showed double the increase in ADHD from 2003 to 2007 relative to other states [29]. Some state legislatures became concerned that schools were overly influential in mental health diagnoses and decisions about psychiatric medications for students. Fourteen states enacted Child Psychiatric Drug Laws (CPDL) which instructed public school boards to prohibit school personnel from recommending that a child take a psychotropic medication, mandate that a child take a psychotropic medication as a condition of enrollment, or use a parent’s refusal to medicate a child as the single basis for a neglect accusation [29]. Stigma against persons with ADHD can result from the public’s uncertainty about the reliability and validity of the diagnosis [30] or that this disorder could result from maternal drinking or smoking [24, 31] and might discourage diagnoses. However, health care spending for children and adolescents in the US for ADHD ($20.6 Billion in 2013) exceeded costs for asthma, depression and anxiety, combined [32]. Medicaid provides health care for one-fifth of the US population and there is substantial autonomy in state Medicaid policies regarding pharmacological ADHD treatments [33]. Approximately one in twenty Hispanics, age 6–17, with Medicaid coverage were treated for ADHD versus one in six non-Hispanic whites [34].

The US accounts for <5% of the world’s population but 83.1% of the global volume of ADHD medications [35]. Stimulants are also employed for non-ADHD indications (Table 1) which may also be expanding. The US Territories are often overlooked in pharmacoepidemiological research. Therefore, the first objective was to expand upon earlier pharmacoepidemiological reports [1–3, 10, 36–41] and evaluate any changes in stimulants in the US in the last decade. Our second objective was to characterize any regional or ethnic differences in the use of these agents.

## Materials and methods

### Procedures

Data was obtained from the Automation of Reports and Consolidated Orders System (ARCOS) for amphetamine, methylphenidate (D, L, and DL isomers), lisdexamfetamine, and D-methamphetamine weights for each year from 2006 to 2016. Manufacturers and distributors are legally required to report on their controlled substances transactions to ARCOS. Non-controlled prescription drugs (e.g. bupropion, atomoxetine) or Schedule IV agents (modafinil) are not reported to ARCOS. This information was publically available at [42]. The greater comprehensiveness of ARCOS for reporting controlled substances relative to a state Prescription monitoring Program can be found elsewhere [43]. Procedures were approved by the University of New England Institutional Review Board (081417–002).

### Data-analysis

Total weight in metric tons was calculated for each drug per year. The 55 US municipalities were divided into the Western, Midwestern, Southern (including Washington DC), and Northeastern regions and also the Territories (American Samoa, Guam, Puerto Rico, and the Virgin Islands). A population corrected index of each medication was calculated as the weight divided by the state population as estimated by the US American Community Survey (ACS) for 2016. States were ranked on this measure for each agent, expressed as the ratio relative to the lowest state, and exploratory analyses were completed. A crude index of overall stimulant use per capita was calculated as the total weight of all four substances. One-sample z-tests were conducted to determine if any states were significantly different from the state average. As Hispanic ethnicity is associated with lower rates of ADHD diagnoses [1, 36, 40], correlations between the percent Hispanic population in each state according to the 2015 ACS and stimulant use was performed. A comparison among the states that had, and had not, enacted CPDLs [29] was made. If the homogeneity of variance assumption was not met (*p* < .10), then the separate variance t-test was reported. Statistics were run using Systat, version 13.1 and figures were prepared with GraphPad Prism, version 6.07. Variability was expressed as the SEM and a *p* < .05 was considered statistically significant.

## Results

**Fig 1:**
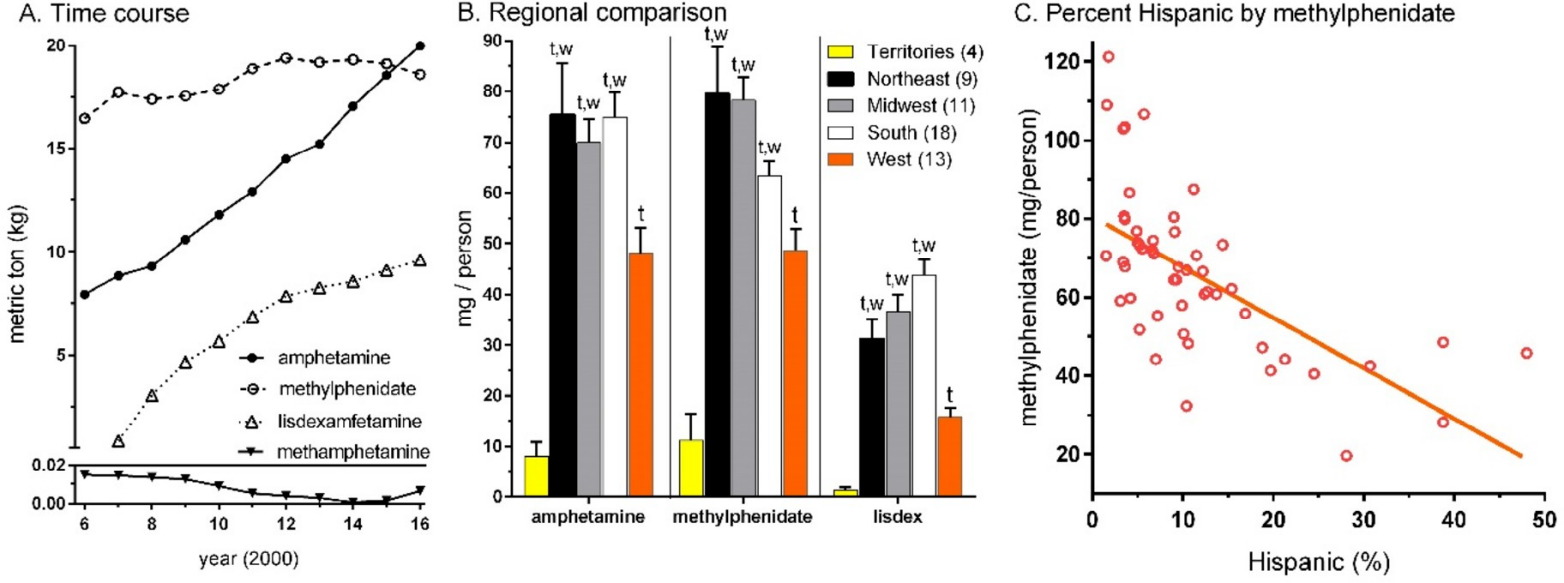
Total weight of amphetamine, methylphenidate, lisdexamfetamine, and methamphetamine in the US and territories from 2006 to 2016. (A). Regional analysis of these drugs in 2016. Lixdex: lisdexamfetamine. ^t^*p* ≤ .001 versus the US Territories; ^w^*p* < .05 versus the western states (B). Scatterplot depicting moderate association (R^2^ = 0.40) between percent of the state comprised of Hispanic citizens according to the 2015 US Census and population corrected use of methylphenidate (C).

Fig 1A shows that amphetamine use increased 2.5 fold from 2006 (7.94 metric tons) to 2016 (19.97). Use of lisdexamfetamine, approved in 2007, steadily increased each year (9.60 in 2016). Methylphenidate consumption was lower in 2006 (16.46), peaked in 2012 (19.39) and declined in the past two years (18.60 in 2016). Amphetamine use overtook methylphenidate in 2016. From 2015 to 2016, there were increases in the average per municipality for amphetamine (+7.6%, *p* ≤ .0005) and lisdexamfetamine (+5.5%, *p* ≤ .0005) but a reduction in methylphenidate (-2.7%, *p* ≤ .0005). Methamphetamine consumption, although very uncommon relative to other agents, increased four-fold from 2015 (26.4 ± 5.6 g) to 2016 (117.8 ± 23.2 g, *p* < .0005).

The net total of the four stimulants doubled from 2006 (24.42) to 2016 (48.18). The areas with the largest increases in total weights between 2015 and 2016 were Florida (+5.8%), Idaho (+6.1%), Utah (+6.5%), Nevada (+7.2%), and Washington DC (+7.7%). Seven states showed net reductions including Maine (-0.2%), Pennsylvania (-0.8%), New Mexico (-0.9%), North Dakota (-1.2%), Ohio (-1.3%), West Virginia (-2.6%), and South Dakota (-5.1%).

**Fig 2:**
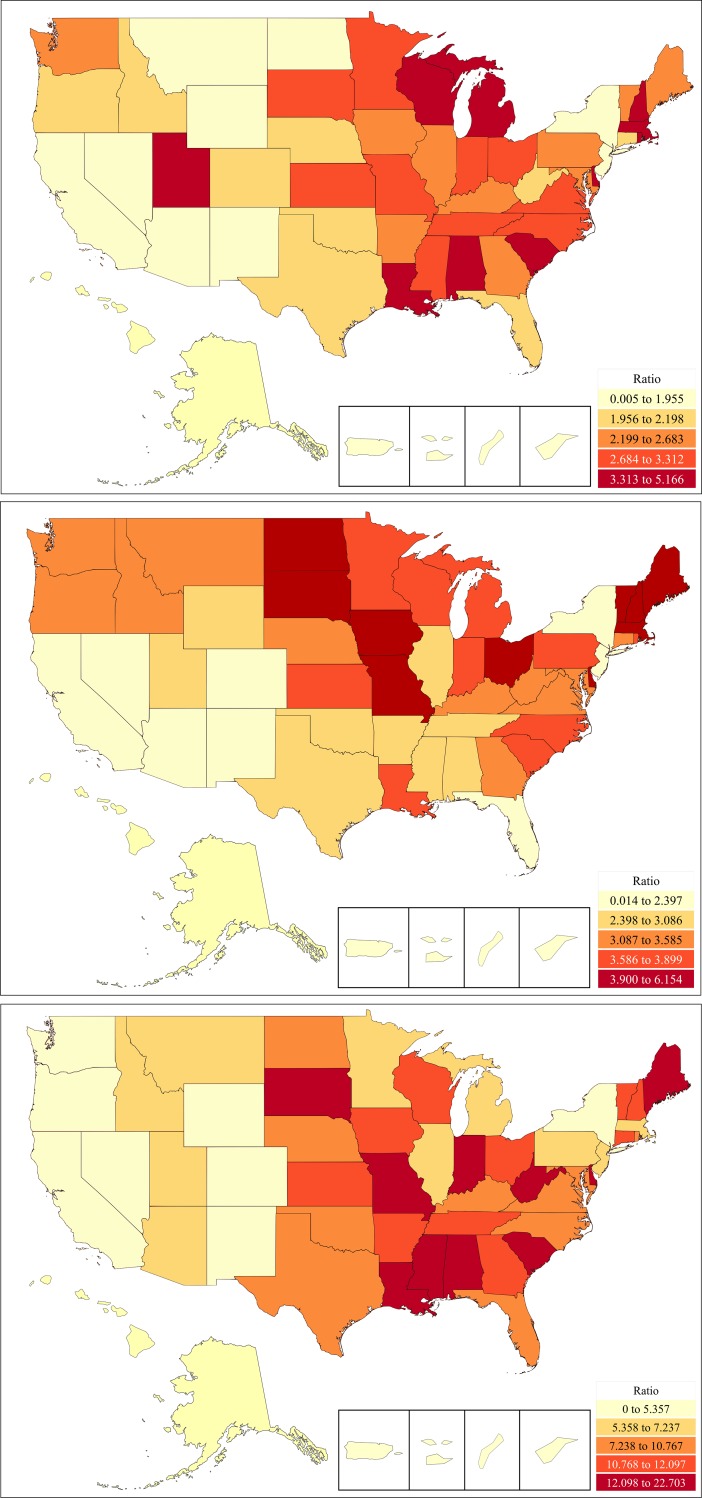
**Heat map of per capita of amphetamine (A, top), methylphenidate (B, middle), or lisdexamfetamine (C, bottom) as reported to the Drug Enforcement Administration in 2016.** Ratio relative to the lowest state (amphetamine = 25.52 mg/capita, methylphenidate = 19.70 mg/capita, lisdexamfetamine = 3.63 mg/capita).

There were sizable differences in population adjusted stimulant use in 2016. There were five-fold consumption differences between the highest versus lowest states for amphetamine use (Fig 2A and Fig A in S1 Fig). Louisiana (*p* < .02) and Rhode Island (*p* < .006) were elevated relative to the national average (67.1 ± 3.3). There was a six-fold use difference between Vermont versus Nevada for methylphenidate (Fig 2B and Fig B S1 Fig). Relative to the state average (65.7 ± 2.9), Iowa, Maine, and Vermont were significantly higher and Nevada lower in consumption. A 22.7 fold difference was found between Louisiana and Hawaii for lisdexamfetamine (Fig 2C and Fig C in S1 Fig) use. Louisiana’s consumption was elevated relative to the other states (32.9 ± 3.6).

Fig 1B depicts pronounced regional differences in the mean population corrected amounts in 2016. Volumes were consistently lower in the Territories relative to other regions. Lisdexamfetamine in the Territories was less-than one-twentieth (4.3%) quantities in the Northeast and a similar pattern was observed for amphetamine (10.7%) and methylphenidate (14.0%). Relative to the national average (165.7 ± 6.9), Louisiana (279.0) was significantly higher and Nevada and Hawaii were significantly lower for total stimulants per capita (S2 Fig).

States with more Hispanic citizens had lower per capita volumes of amphetamine (*r*(49) = -0.43, *p* ≤ .002, S3A Fig), lisdexamphetamine (*r*(49) = -0.49, *p* ≤ .0005, S3B Fig), and methylphenidate (*r*(49) = -0.63, *p* ≤ .0005, Fig 1C). The presence of a CPDL did not appreciably impact the per capita volume of amphetamine or lisdexamfetamine. States with a CPDL had 12.0 mg less of methylphenidate than those without (57.0 ± 5.2 versus 68.9 ± 3.3) but this difference was not statistically significant (*p* = .063).

## Discussion

The primary objective was to examine the temporal pattern of stimulants in the US. This topic is important because these Schedule II drugs have considerable misuse potential and some adverse effects. Use patterns reflect a combination of factors including the broadening ADHD diagnostic criteria and use of the DSM-5 [8] in the US, rates of off-label use for other indications such as obesity and narcolepsy, limited availability of non-pharmacological, but evidence-based, therapies like behavioral parent training and behavioral classroom management [12, 21], patent expirations, the convenience of once daily dosing, and the socio-legal [7, 29], economic [35, 44], or cultural characteristics [15, 34, 45] that influence decision-making about the relative risks and harms for these agents. ADHD diagnoses increased among all children by 42% from 2003 to 2011 [1] and by 83% from 2001 to 2010 among low-income US children [46]. ADHD prevalence may be anticipated to increase further following the 2013 publicatoin of DSM-5 which is more inclusive of adult-ADHD [8]. The US population grew by eight-percent from 2006 to 2016 while methylphenidate use increased 13.0%, amphetamine use doubled and lisdexamfetamine use showed pronounced gains. Total use of these four stimulants doubled. These elevations extend upon other research [1, 9–10, 15, 34]. However, the expansion of the BMI among US children [47] may have contributed via increased stimulant treatments for obesity or BED. Alternatively, the decline in school time physical activity (eg. recess and gym class) may have contributed to classroom hyperactivity and stimulant prescribing requests from schools. While stimulants may offer substantial benefits to children, for example those in [48], it is also important to recognize that they are not benign, and that there is substantial diversion and misuse potential for these Schedule II substances [49–53].

The individual and societal cost for pharmacotherapies to improve attention is not insignificant [35, 54]. US expenditures for ADHD medications increased 594% between 1994 and 2003 [54]. Commercial insurance plans spent more per patient for medications for attention disorders than for asthma, heart disease/hypertension, or dyslipidemia [44]. Health care spending for ADHD in 2013 was estimated at $20.6 Billion (equivalent to the mid-career salary for 230,000 psychologists, 365,000 teachers, or 827,000 teacher’s aids). The ubiquity of these agents may warrant additional fiscal consideration if current trends continue.

The second objective was to identify any regional differences [2]. According to parental reports, Nevada ranked lowest in the US for the percent of children with ADHD (4.2%) and also was lowest for the percent of children currently receiving ADHD medications [1]. Therefore, our finding that Nevada ranked among the lowest three states for amphetamine, methylphenidate, and lisdexamfetamine use was unsurprising [36]. Vermont and Maine, two-states with the highest median ages, led the country for methylphenidate consumption. These states may be more readily using this agent for adult-ADHD [55]. Alternatively, these states have limited diversity and whites show the highest rates of drug treatment for ADHD [34]. Pharmacoeconomic disparities could account for the differences between the Territories and the rest of the US, particularly for more expensive agents although lower rates of ADHD diagnoses [3] are likely also an important contributing factor. The vast majority (92.8%) of Puerto Rican children with ADHD, despite having insurance, had not received any medications and three-fifths (60.0%) had not received any psychoeducational interventions [56]. Interviews revealed concerns about anorexia and addiction [56]. Nationwide, the diversion of stimulants, specifically amphetamine and methylphenidate, for non-medical use was considerable [50].

Approximately one-third of states enacted CPDL between 2001 and 2009 [29]. Data was only weakly suggestive that this legislation was impactful as states with these laws had a 17.4% lower per capita methylphenidate use. However, this observation is limited by the fact that non-controlled medication therapies for ADHD such as atomoxetine, whose use from 2004 to 2010 decreased by ≥ 50% [10], were not reported to ARCOS. The contribution of any state law to stimulant medication use may have been obscured by the federal *Individuals with Disabilities Education Improvement Act* of 2004 which prohibited educational personnel from requiring a child, as a condition of attending school, to receive a controlled substance.

These findings are important when placed within an international [20, 21, 41, 57–60] and historical [35, 59] context. The 1997 clarification by the US FDA broadcast regulations opened up the potential for Schedule II drugs to be actively advertised directly to consumers [61]. There were pronounced increases in ADHD during the 2003, 2007, and 2011 waves of the well-powered and representative samples obtained in the National Survey of Children’s Health [1,2]. Among 55 countries that had adopted the use of ADHD medications in 2003, the US (<5% of the world’s population) was responsible for > 92% of the world spending on these agents [35]. Although the methodology across countries differs, the 2011 report from the US CDC that nearly 1 in 5 high-school boys and 1 in 11 high school girls had been diagnosed with ADHD would clearly position these rates as among the highest in the world [58, S2 Table]. The retail pharmaceutical spending in the US, per capita, is almost three-fold greater than the UK [59]. Although a portion of this difference is likely due to greater costs per prescription, the greater utilization of some classes of neuropsychiatric agents (stimulants, antipsychotics, opioids) may warrant further pharmacoeconomic consideration.

There are at least three key factors which may account for the high rates of ADHD, and ADHD medication use, in the US [35] relative to other countries [41, S2 Table). First, the ICD criteria are much more conservative than the DSM [20]. Further, each revision of the DSM may have contributed to this pattern [39, 62]. A meta-analysis of reports from China identified a significantly lower prevalence when DSM-III criteria were employed relative to DSM-IV or DSM-5 [41]. Given the subjective nature of information relied upon for ADHD diagnosis, it is unfortunate that efforts to detect feigned ADHD symptoms in adults are only at an early research stage [63–69]. Second, the US is unusual that it allows for direct to consumer advertising of prescription pharmaceuticals including controlled substances [61]. The US has included provisions that were intended to favor direct to consumer advertising of pharmaceuticals in trade agreements with Australia, South Korea, and other countries [70]. Easy accessibility of online disease checklists or screening forms may function as promotional devices [20] or as informational resources to facilitate malignering. Third, patients and diagnosticians respond to financial incentives. The US Supreme Court decision of *Sullivan v*. *Zebley* resulted in the inclusion of ADHD for Supplemental Security Income (SSI) benefits in 1990. There was a three-fold increase in SSI benefits for children between 1989 and 1995. More recently, of the $10.5 Billion expended by the childhood SSI program in 2013, almost two-thirds (65.0%) was for mental disorders of which ADHD was the most common allowance (21.9% or $1.5 Billion, [71].

Further progress in understanding the pathophysiology of ADHD [22] and the development of laboratory measures that could be incorporated into diagnostic criteria [8] would aid in the sensitivity and specificity of diagnosis. The question of whether there is a pre-existing biochemical imbalance in ADHD is contentious [72]. Interestingly, non-human research has found that long-term blockade of the dopamine transporter results in adaptive changes in this protein [73–75]. A two-week course with a clinically relevant (2 mg/kg) dose of methylphenidate reduced the dopamine transporters in the rat striatum by almost 50% [73]. A meta-analysis of Positron Emission Tomography investigations in humans concluded that striatal dopamine transporter density was also changed by psychostimulant exposure [72].

There are some strengths and limitations to the ARCOS dataset and this report. ARCOS monitors the quantities by weight of select controlled substances from manufacture to sale. The total weight of prescription stimulants approximately doubled between 2006 and 2016. The extended time-frame, objectivity, and comprehensive examination of the entire US, and US Territories, independent of type of insurance coverage, are assets of this investigation which extends upon prior work [1,9,10,15]. ARCOS is arguably the gold-standard for broad coverage of select controlled substances for pharmacoepidemiology investigations in the US [43], and this is the first investigation using ARCOS data for stimulants. Perhaps the largest caveat with ARCOS is that age of recipients, indication, formulation, or daily dose for these agents are not contained in this data source. Examination of health insurance plans determined that three-fifths of youths (≤19) prescribed stimulants had an ADHD diagnosis versus less than half (45.5%) of adults [76]. Additional study is needed to quantify the portion of this stimulant increase that is due to ADHD versus obesity, narcolepsy, depression, non-ADHD cognitive enhancement, or other off-label use [77]. ARCOS does not report on the non-stimulants (atomoxetine, clonidine, guanfacine, bupropion) that are non-first-line agents for ADHD and does not distinguish between different stimulant formulations. This study does not directly address whether ADHD is being overdiagnosed [62,78–80], misdiagnosed, or underdiagnosed [81] or whether stimulants are being over-utilized [82] or under-utilized [56,81]. No inferences about the over or under-treatment of ADHD among specific ethnicities or states should be made without additional information about the utilization of non-pharmacological services. Further study will be necessary to characterize which ages (preschool, child, adolescent, or adult) and which indications (ADHD, obesity, BED) are responsible for the increases in amphetamine and lisdexamfetamine. Comparison of the weights of different stimulants should only be made while recognizing that these agents have different potencies [11, Table 1]. ARCOS also does not provide direct insight into how many children and adolescents are receiving multiple pharmacotherapies for ADHD [83]. Over one-fifth of the market share for drugs on the online black market (Silk Road) was for prescription stimulants [84]. Importantly, ARCOS may capture a larger portion of controlled substances which were subsequently diverted for non-medical purposes [50] than other data sources. Examination of other sources [54] will be necessary to monitor formulations (e.g. a sweet chewable form of amphetamine) which may have a heightened diversion potential.

In conclusion, this report identified increases in methylphenidate, amphetamine and lisdexamfetamine use in the US over the last decade. The US Territories, western US, and states with larger Hispanic populations had lower stimulant use. Additional study is necessary to characterize the sociocultural and economic factors responsible for the pronounced regional variations in stimulant use in the US.

## Supporting information

S1 Fig Doubling in total weight (metric tons) of amphetamine, methylphenidate, lisdexamfetamine, and methamphetamine as reported to the Drug Enforcement Administration for the United States and Territories from 2006 to 2016. (DOCX)

S2 Fig Per capita (mg/person) of amphetamine, methylphenidate, and lisdexamfetamine as reported to the Drug Enforcement Administration in 2016, ranked in the United States and US Territories. (TIFF)

S3 Fig Heat map of per capita (mg/person) of stimulants (amphetamine, methylphenidate, lisdexamfetamine, and methamphetamine) as reported to the Drug Enforcement Administration in 2016 (A). Per capita (mg/person) of stimulants (amphetamine, methylphenidate, lisdexamfetamine, and methamphetamine) as reported to the Drug Enforcement Administration in 2016, ranked in the United States and US Territories (B).
(TIFF)

S4 Fig Scatterplots with linear regression showing that states with more Hispanic citizens had lower per capita volumes of amphetamine (A, *r*(49) = -0.43, *p* ≤ .002) and lisdexamphetamine (B, *r*(49) = -0.49, *p* ≤ .0005).
(DOCX)

S1 Table Ten highest and ten lowest states and the US Territories for the mg per person of amphetamine, methylphenidate, and lisdexamfetamine as reported to the US Drug Enforcement Agency’s Automation of Reports and Consolidated Orders System for 2016. ^P^state with a psychotropic medication law. ^H^state among the top ten in the country for highest percent Hispanic population.
(DOCX)

S2 Table Global comparison of ADHD diagnosis and ADHD medication prevalence. MPH: methyphenidate; ^E^estimated.
(DOCX)
