# Supplementary material for: Trends in use of prescription stimulants in the United States and Territories, 2006 to 2016
Source: PLoS One. 2018 Nov 28;13(11):e0206100. doi: 10.1371/journal.pone.0206100 (PMC6261411; doi:10.1371/journal.pone.0206100)
Supplement: S1 Fig — (DOCX) [file pone.0206100.s001.docx]

**S1 Fig. Doubling in total weight (metric tons) of amphetamine, methylphenidate, lisdexamfetamine, and methamphetamine as reported to the Drug Enforcement Administration for the United States and Territories from 2006 to 2016.**

*
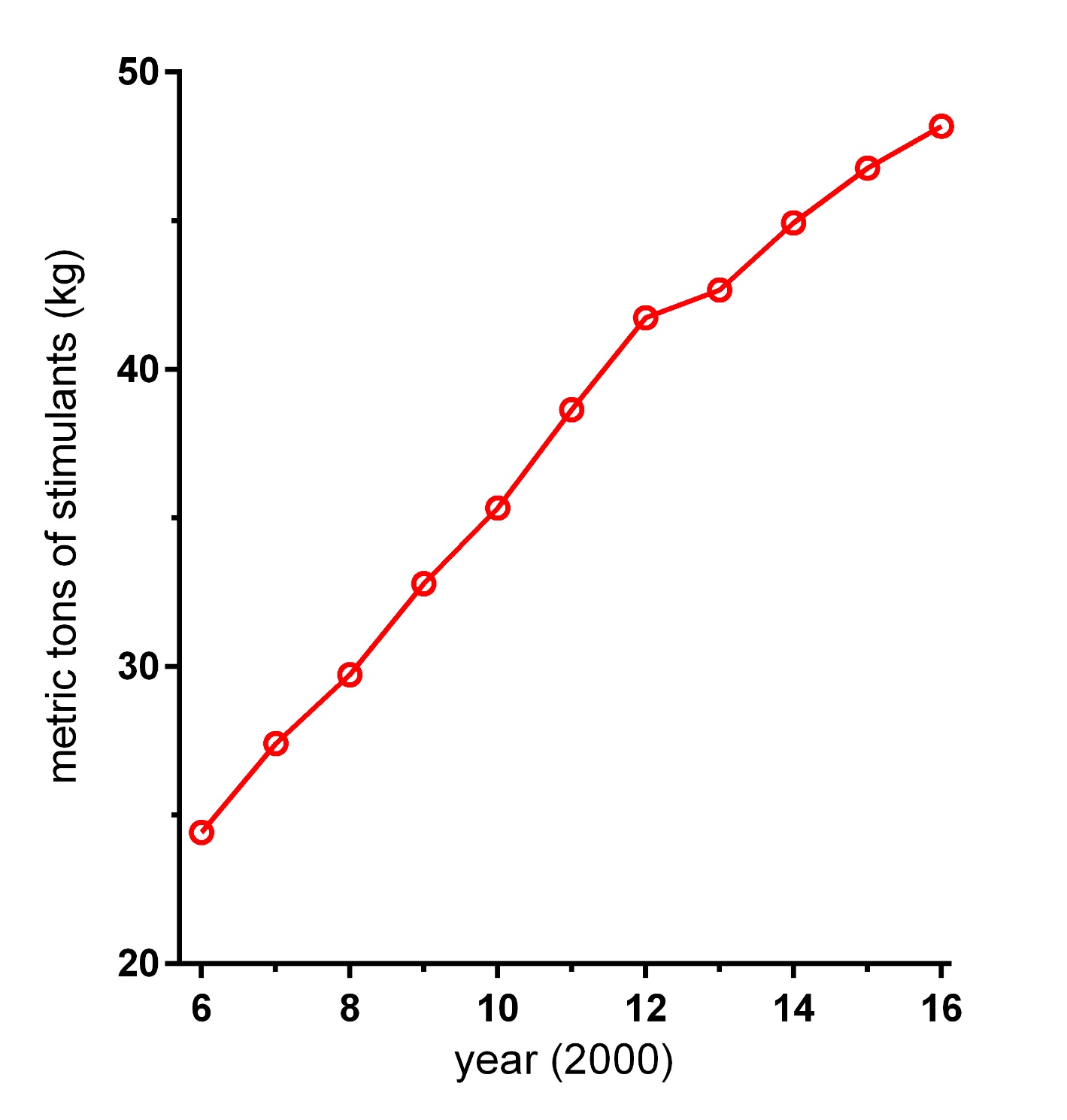
*
