# Supplementary figures and images for: Trends in use of prescription stimulants in the United States and Territories, 2006 to 2016
Source: PLoS One. 2018 Nov 28;13(11):e0206100. doi: 10.1371/journal.pone.0206100 (PMC6261411; doi:10.1371/journal.pone.0206100)

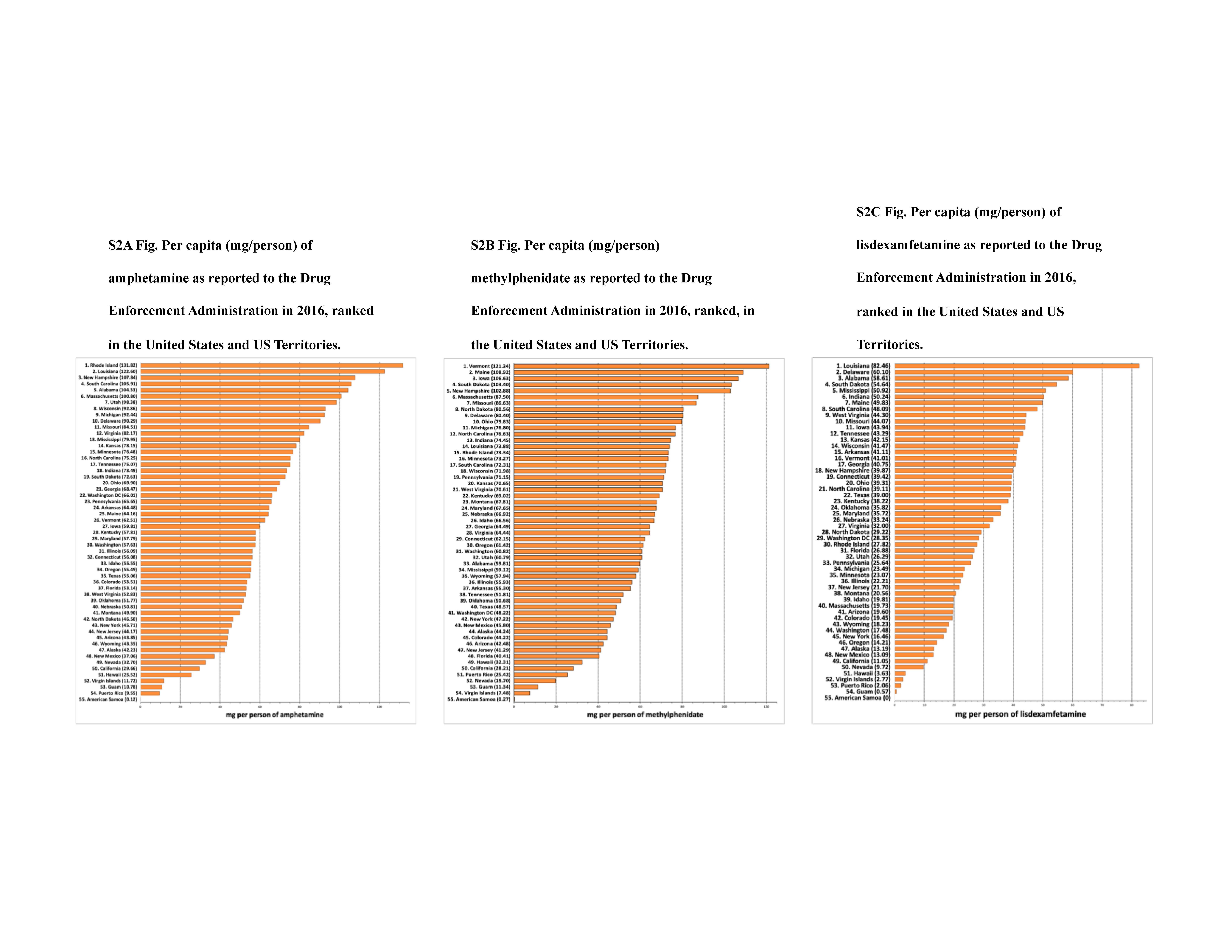

Supplement: S2 Fig — (TIFF) [file pone.0206100.s002.tiff]

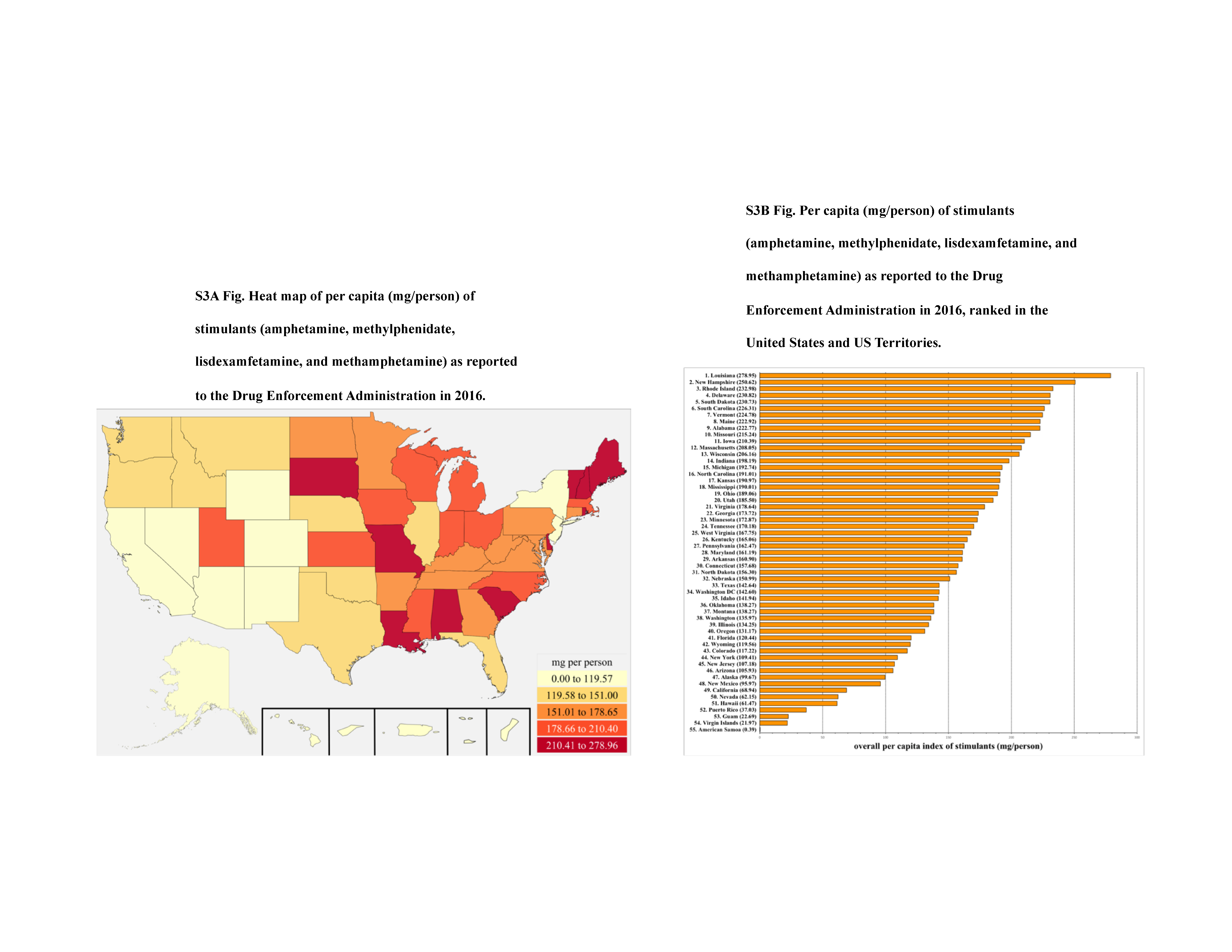

Supplement: S3 Fig — Heat map of per capita (mg/person) of stimulants (amphetamine, methylphenidate, lisdexamfetamine, and methamphetamine) as reported to the Drug Enforcement Administration in 2016 (A). Per capita (mg/person) of stimulants (amphetamine, methylphenidate, lisdexamfetamine, and methamphetamine) as reported to the Drug Enforcement Administration in 2016, ranked in the United States and US Territories (B). (TIFF) [file pone.0206100.s003.tiff]
