# Supplementary material for: Trends in use of prescription stimulants in the United States and Territories, 2006 to 2016
Source: PLoS One. 2018 Nov 28;13(11):e0206100. doi: 10.1371/journal.pone.0206100 (PMC6261411; doi:10.1371/journal.pone.0206100)

**S4 Fig**. **Scatterplots with linear regression showing that states with more Hispanic citizens had lower per capita volumes of amphetamine (A, *r*(49) = -0.43, *p* < .002) and lisdexamphetamine (B, *r*(49) = -0.49, *p* < .0005).**


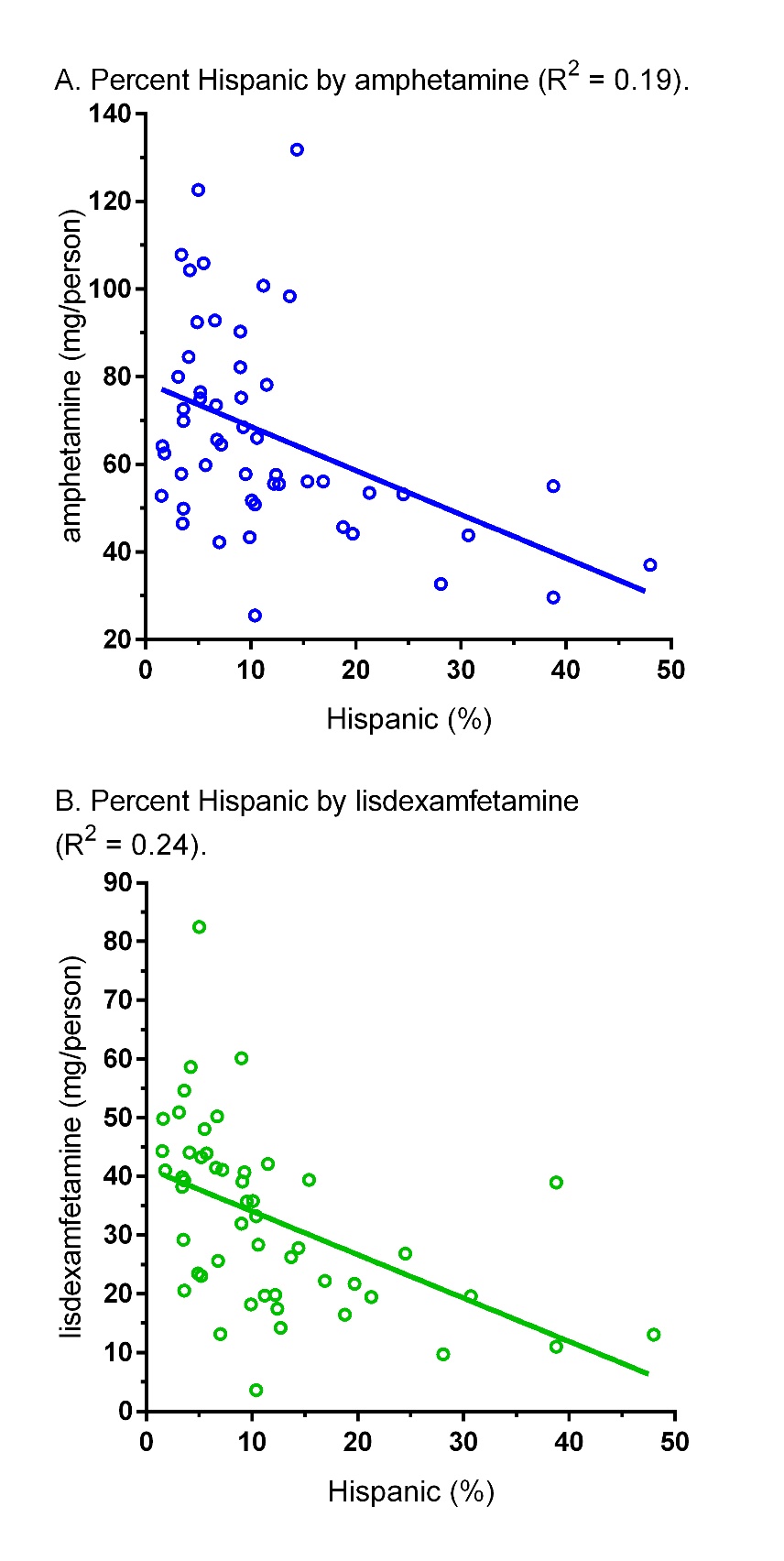

Supplement: S4 Fig — Scatterplots with linear regression showing that states with more Hispanic citizens had lower per capita volumes of amphetamine (A, r(49) = -0.43, p ≤ .002) and lisdexamphetamine (B, r(49) = -0.49, p ≤ .0005). (DOCX) [file pone.0206100.s004.docx]
