# Supplementary material for: Trends in use of prescription stimulants in the United States and Territories, 2006 to 2016
Source: PLoS One. 2018 Nov 28;13(11):e0206100. doi: 10.1371/journal.pone.0206100 (PMC6261411; doi:10.1371/journal.pone.0206100)
Supplement: S1 Table — Pstate with a psychotropic medication law. Hstate among the top ten in the country for highest percent Hispanic population. (DOCX) [file pone.0206100.s005.docx]

**S1 Table**. **Ten highest and ten lowest states and the US Territories for the mg per person of amphetamine, methylphenidate, and lisdexamfetamine as reported to the US Drug Enforcement Agency’s Automation of Reports and Consolidated Orders System for 2016.** _________________________________________________________________________________________________________

State Rank Amphetamine Methylphenidate Lisdexamfetamine

#1 Rhode Island: 131.8 Vermont: 121.2 Louisiana: 82.5^P^

#2 Louisiana: 122.6^P^ Maine: 108.9 Delaware: 60.1

#3 New Hampshire: 107.8^P^ Iowa: 106.6 Alabama: 58.6

#4 South Carolina: 105.9 South Dakota: 103.4 South Dakota: 54.6

#5 Alabama: 104.3 New Hampshire: 102.9^P^ Mississippi: 50.9

#6 Massachusetts: 100.8 Massachusetts: 87.5 Indiana: 50.2

#7 Utah: 98.4^P^ Missouri: 86.6 Maine: 49.8

#8 Wisconsin: 92.9 North Dakota: 80.6 South Carolina: 48.1

#9 Michigan: 92.4 Delaware: 80.4 West Virginia: 44.3

#10 Delaware: 90.3 Ohio: 79.8 Missouri: 44.1

----------------------------------------------------------------------------------------------------------------------------------------------------------------

State average (+SEM) 67.1 + 3.3 65.7 + 2.9 32.9 + 2.1

----------------------------------------------------------------------------------------------------------------------------------------------------------------

#41 Montana: 49.9 Washington DC: 48.2 Arizona: 19.6^H^

#42 North Dakota: 46.5 New York: 47.2 Colorado: 19.5^P^

#43 New York: 45.7 New Mexico: 45.8^H^ Wyoming: 18.2

#44 New Jersey: 44.2^H^ Alaska: 44.2^P^ Washington: 17.5

#45 Arizona: 43.8^PH^ Colorado: 44.2^PH^ New York: 16.5

#46 Wyoming: 43.3 Arizona: 42.5^H^ Oregon: 14.2^P^

#47 Alaska: 42.2^P^ New Jersey: 41.3^H^ Alaska: 13.2

#48 New Mexico: 37.1^H^ Florida: 40.4^PH^ New Mexico: 13.1^H^

#49 Nevada: 32.7^PH^ Hawaii: 32.3 California: 11.0^H^

#50 California: 29.7^H^ California: 28.2^H^ Nevada: 9.7^PH^

#51 Hawaii: 25.5 Nevada: 19.7^PH^ Hawaii: 3.6

Territory Rank

#1 Virgin Islands 11.7 Puerto Rico 25.4 Virgin Islands 2.8

#2 Guam 10.8 Guam 11.3 Puerto Rico 2.1

#3 Puerto Rico 9.5 Virgin Islands 7.5 Guam 0.6

#4 American Samoa 0.1 American Samoa 0.3 American Samoa 0.0

^P^state with a psychotropic medication law. ^H^state among the top ten in the country for highest percent Hispanic population.
