# Supplementary material for: Trends in use of prescription stimulants in the United States and Territories, 2006 to 2016
Source: PLoS One. 2018 Nov 28;13(11):e0206100. doi: 10.1371/journal.pone.0206100 (PMC6261411; doi:10.1371/journal.pone.0206100)
Supplement: S2 Table — MPH: methyphenidate; Eestimated. (DOCX) [file pone.0206100.s006.docx]

**S2 Table. Global comparison of ADHD diagnosis and ADHD medication prevalence.**

___________________________________________________________________________________________________________________________

Location Year Sample (Age) % ADHD (Age) % Medication Other Citation

Canada 1994 12,595 (3-9) 1.3 [64]

2006 14,565 (3-9) 2.1 [64]

China varied 275,502 ( <18) 6.3 Inattentive subtype most common [66]

Denmark 2005 1,203,817 (0-19) 0.4 MPH = 98.9% [15]

2012 1,203,817 (0-19) 1.5 MPH = 81.3% [15]

France 2003 374,221 (6-18) 0.11 MPH = 100.0% [85]

2005 430,150 (6-18) 0.18 MPH = 100.0% [85]

Germany 2009 24 million (0-69) 5.0 (0-17) MPH is most common [62]

2014 24 million (0-69) 6.1 (0-17) MPH is most common [62]

2014 24 million (0-69) 0.2% (18-69) MPH is most common [62]

2014 24 million (0-69) 0.4% (18-69) MPH is most common [62]

Hong Kong 2001 1.4 million (3-19) 0.07 MPH = 100% [84]

2013 1.0 million (3-19) 1.03 MPH = 95.0% [84]

Italy 2011 6,183 (5-15) 3.1% 0.00 56.8% ADHD combined subtype [86]

Netherlands 2005 131,954 (0-19) 1.8 MPH = 97.8% [15]

2012 131,954 (0-19) 3.9 MPH = 94.2% [15]

Sweden 2006 9.1 million (0–100) 0.11 0.88^E^ MPH = 83.5% [87]

2011 9.5 million (0-100) 0.48 0.38^E^ MPH = 83.5% [87]

United Kingdom 2005 827,906 (0-19) 0.3 MPH = 90.4% [15]

2012 827,906 (0-19) 0.5 MPH = 86.6% [15]

US 2003 102,353 (4-17) 7.8 4.3 lower % in the Western US [88]

2007 91,642 (4-17) 9.5 4.8 lower % among Hispanics [2]

2011 76,015 (4-17) 11.0 6.1 lower % among Hispanics [1]

________________________________________________________________________________________________________________________

MPH: methyphenidate; ^E^estimated.

**S References**

[85] Knellwolf AL, Deligne J, Chiarotti F, Auleley GR, Palmieri S, Boisgard CB, et al. Prevalence and patterns of methylphenidate use in French children and adolescents. *Eur J Clin Pharmacol* 2008; 64:311-7.

[86] Bianchini R, Postorino V, Grasso R, Santoro B, Migliore S, Burlò C, et al. Prevalence of ADHD in a sample of Italian students: a population-based study*. Res Dev Disabil*. 2013; 34:2543-50.

[87] Giacobini M, Medin E, Ahnemark E, Russo LJ, Carlqvist P. Prevalence, patient characteristics, and pharmacological treatment of children, adolescents, and adults diagnosed with ADHD in Sweden. *J Atten Disord* 2018; 22:3-13.

[88] Centers for Disease Control and Prevention (CDC). Mental health in the United States. Prevalence of diagnosis and medication treatment for attention-deficit/hyperactivity disorder--United States, 2003. *MMWR Morb Mortal Wkly Rep* 2005; 54:842-7.
